# Supplementary material for: Development of a nomogram for overall survival prediction in primary upper lobe lung cancer patients: A SEER population-based analysis
Source: PLoS One. 2025 Apr 29;20(4):e0321955. doi: 10.1371/journal.pone.0321955 (PMC12040116; doi:10.1371/journal.pone.0321955)
Supplement: Attachment — Submitted filename: Major revision.docx [file pone.0321955.s001.docx]

**Development of a nomogram for overall survival prediction in primary upper lobe lung cancer patients: a SEER population-based analysis**

Wenze Yu, Lu Long, Qizhuo Hou, Bin Yi

The study investigates lung cancer patients with a primary tumor in the upper lobe using data from the SEER program (2010–2017). It aims to identify prognostic factors of lung cancer in the upper lobe and to establish an effective nomogram for individualized overall survival prediction. The manuscript is well-structured and covers critical aspects of lung cancer prognosis, including : demographic characteristics, histology, staging, and metastasis. But it could benefit from some modifications to enhance its scientific and clinical robustness:

Major points:

- Crucial parts of the manuscript are the development and validation of the nomogram are. However, some of the descriptions of the figures could be clearer. For example, you state, “The survival probability of 2-year and 3-years were shown in Fig 5,” but it would be better to describe how the variables were weighted and how the nomogram can be practically used.

- When discussing the calibration curves (Fig. 7), elaborate on what the results of the calibration plot suggest about the performance of your model.

- In addition to the limitations already mentioned in your study, such as the lack of data on treatment modalities and the need for external validation of the model, the exclusive use of clinical variables may not fully capture the biological and molecular complexity of the disease. Genetic and molecular biomarkers are becoming increasingly important in the prognosis and therapeutic decision-making for lung cancer. It would be beneficial to integrate molecular information (e.g., gene mutations, expression of specific biomarkers such as EGFR, ALK, or PD-L1). This approach could enhance the accuracy of prognosis. You could highlight this aspect.

- The discussion touches on several interesting findings, such as the association between primary tumor site and survival. However, the mechanism behind the poorer prognosis for patients with upper lobe lung cancer should be discussed in more depth. Are upper lobe tumors more likely to spread to other organs (e.g., brain, bones) compared to other lobes? Are there anatomical or biological factors (e.g., proximity to larger blood vessels, lymphatic drainage) that may explain this?

-The discussion could emphasize the prospective application in clinical practice of the nomogram developed. For example, could this nomogram be used to guide treatment decisions? Is it accurate enough to influence decisions on the timing of surgery or the need for adjuvant therapies?

Minor points:

- Ensure consistency in terms of abbreviations (e.g., "Mets at DX" for metastasis at diagnosis). In some instances, it’s abbreviated and in others not.

- In the statistical analysis section, it might be helpful to spell out abbreviations like HR (hazard ratio) when first introduced.
